# The effects of transition to technician‐delivered telehealth ABA treatment during the COVID‐19 crisis: A preliminary analysis

**DOI:** 10.1002/jaba.803

**Published:** 2020-12-28

**Authors:** Joy S. Pollard, Linda A. LeBlanc, Christan A. Griffin, Joseph M. Baker

**Affiliations:** ^1^ Behavior Change Institute; ^2^ Stanford University School of Medicine; ^3^ LeBlanc Behavioral Consulting

**Keywords:** autism, direct services, telehealth

## Abstract

Telehealth delivery of applied behavior analysis treatment has focused on supervision or staff and parent training, rather than the direct delivery of treatment to clients. The novel coronavirus (COVID‐19) crisis had the potential to significantly disrupt access to direct treatment for individuals with autism. We report a sample of 17 cases that transitioned from in‐person to telehealth delivery of treatment when shelter‐in‐place orders were issued. Of these cases, 76% of participants transitioned to technician‐delivered telehealth services whereas the rest transitioned to a caregiver‐implemented telehealth model. Participants continued to access a similar dosage of treatment hours per week in spite of the treatment model transition (in‐person *M* = 12; telehealth *M* = 11) and maintained or improved correct independent responding across all targets from in‐person treatment (*M* = 75%) to telehealth treatment (*M* = 80%). These findings provide initial evidence that some clients with autism benefit from technician‐delivered telehealth services.

Telehealth is defined by the Health Resources and Services Administration as the “use of electronic information and telecommunication technologies to support and promote long‐distance clinical health care, patient and professional health‐related education, public health and health administration” (Health Information Technology, 2017, para 1). Importantly, telehealth is a means of delivering health care services rather than a distinct or separate healthcare service. This unique means of service delivery allows providers to deliver the healthcare service directly to the client without requiring the client to travel to receive care. Provider travel time to deliver care is also reduced, thus overcoming barriers such as geographic isolation and limited access to qualified professionals (Lerman et al., 2020; Pollard et al., 2017; Rispoli & Machalicek, 2020).

Telehealth has been employed for almost two decades in the delivery of applied behavior analytic (ABA) assessment and treatment services (Tomlinson et al., 2018). Individuals with autism spectrum disorders (ASD) and intellectual and developmental disabilities (IDD) have benefited from the use of telehealth to train caregivers and staff in the assessment and delivery of ABA services (Barretto et al., 2006; Boisvert et al., 2010; Ferguson et al., 2019; Fisher et al., 2014; Lindgren et al., 2016; Tomlinson et al., 2018; Unholz‐Bowden et al., 2020; Vismara et al., 2009). A growing scientific evidence base indicates that some treatment services can be delivered directly to clients via telehealth using synchronous (i.e., real‐time) videoconferencing technology (Ferguson et al., 2020; Myers et al., 2017; Pellegrino & DiGennaro‐Reed, 2020) and that parents and staff can be trained via telehealth to deliver services to clients in‐person (Bearss et al., 2018; Benson et al., 2018; Higgins et al., 2017; Monlux et al., 2019; Suess et al., 2014; Unholz‐Bowden et al., 2020).

The most common version of telehealth services represented in the published literature is a caregiver telehealth coaching model, which involves a clinician providing training via synchronous videoconferencing to another person (e.g., parent, teacher, technician) at a distant site to deliver services in‐person to the client (Council of Autism Service Providers [CASP], 2020a; Lerman et al., 2020). This model has been used for functional behavior assessments (Barretto et al., 2006; Benson et al., 2018; Boisvert et al., 2010), preference assessments (Higgins et al., 2017), behavior reduction procedures (Hall et al., 2020; Lindgren et al., 2016; Monlux et al., 2019; Suess et al., 2016; Suess et al., 2020; Unholz‐Bowden et al., 2020; Wacker et al., 2013a; Wacker et al., 2013b), and interventions to build language, social, and daily living skills (Akemoglu et al., 2020; Barkaia et al., 2017; Ferguson et al., 2019; Ingersoll et al., 2016; McLay et al., 2020; Wainer & Ingersoll, 2015). A telehealth model commonly applied in clinical settings is a *partial in‐person telehealth model* whereby ABA services are rendered in‐person by a trained technician, with clinical oversight provided by a behavior analyst via real‐time, synchronous videoconference modality (CASP, 2020b).

Two recent studies explored a *technician‐delivered telehealth service model* of ABA services, during which the individual clients participated independently in session without caregiver support via synchronous videoconferencing technology (Ferguson et al., 2020; Pellegrino & DiGennarro‐Reed, 2020). That is, the participants received all instructions, prompting, and reinforcement from the provider via real‐time videoconferencing technology. Pellegrino and DiGennarro‐Reed (2020) evaluated the efficacy of total task chaining with least‐to‐most prompting delivered via videoconference to two adults with IDD. Both individuals were living semi‐independently in housing for individuals with IDD. The targeted skills (e.g., cooking, managing a budget) were endorsed by the participants as interesting and relevant to their personal goals. The instruction involved vocal and model prompting, and these prompts were all delivered remotely. Both participants met the mastery criterion for each skill in fewer than 15 sessions. Notably, both participants expressed satisfaction with the goals, procedures, and effects of the intervention. Ferguson et al. (2020) used synchronous videoconferencing to teach tact relations to six children diagnosed with ASD, aged 3 years 11 months to 7 years 1 month. The experimenters taught participants in a dyad arrangement using discrete trial teaching procedures. All participants acquired the targeted skills and maintained responses 9 days following training. The results of these studies are promising for the delivery of ABA treatment via synchronous telehealth modalities with both children and adults with ASD and DD.

The novel coronavirus disease (COVID‐19) pandemic and the subsequent containment measures led to unprecedented challenges to continued access to ABA treatment services for individuals with ASD and IDD (CASP, 2020a,b; LeBlanc et al., 2020; Megan, 2020; Wolfram, 2020). Federal, state, and local governments enacted emergency measures in response to the rapid outbreak of COVID‐19 to slow the spread of the disease. The U.S. government issued a national state of emergency and many states followed suit, implementing shelter‐in‐place restrictions. Although the experimental literature on telehealth delivery of direct ABA services is sparse, the abrupt threat to services and risk for in‐person contact during the COVID‐19 crisis created the need, as well as the opportunity, to explore and evaluate the viability of treatment services delivered directly to clients via telehealth.

To that end, the purpose of this study was to conduct an archival analysis of data collected during the transition from in‐person direct services with telehealth clinical direction to a direct intervention telehealth model by an ABA provider agency during the COVID‐19 pandemic. Use of existing data affords researchers the opportunity to study natural events, has the advantage of reducing threats to internal validity such as experimenter bias, and can indicate the generalizability of results. Therefore, we used this archival data to a) examine client progress in skill acquisition programs before and after the transition, b) examine whether various skills were associated with differential success in this direct telehealth model, and 3) stimulate future research on technician‐delivered telehealth service delivery in ABA treatment.

## Method

### *Organization and Provider Information*

Ten Board Certified Behavior Analysts® (BCBAs®) providers from the same ABA organization contributed data to the sample of 17 client participants. That ABA organization had specialized in providing services under a partial in‐person telehealth model for the past 6 years. All BCBA clinicians were trained to provide clinical direction via a synchronous telehealth modality to Registered Behavior Technicians™ (RBTs™) who were delivering in‐person ABA services prior to COVID‐19. The clinicians and the RBTs had been employed for varying lengths of time at the organization (see Table 1 for provider experience information). The BCBAs were certified for an average of 54 months (range, 21 ‐ 101 months) and had worked at the organization providing supervision via synchronous telehealth for an average of 19 months (range, 0 ‐ 63 months). Sixteen RBTs provided direct services to the participants in this study. The technicians were certified for an average of 20 months (range, 1 ‐ 50 months) prior to starting direct services via telehealth. Technicians had experience receiving supervision via synchronous telehealth, but had no prior history with direct service delivery via telehealth.

**Table 1:** *Provider Experience*

Participant	Provider	Duration Certified	Telehealth Experience
1	BCBA	3 years, 9 months	3 months
	RBT	9 months	NA
2	BCBA	1 year, 9 months	4 months
	RBT	1 month	NA
3	BCBA	2 years, 3 months	1 month
	RBT	1 year, 7 months	NA
4	BCBA	8 years, 5 months	10 months
	RBT	4 months	NA
5	BCBA	2 years, 3 months	1 month
	RBT	2 years, 11 months	NA
6	BCBA	2 years, 9 months	1 year, 3 months
	RBT	1 month	NA
7	BCBA	1 year, 9 months	4 months
	RBT	1 month	NA
8	BCBA	3 years, 9 months	3 months
	RBT	9 months	NA
9	BCBA	3 years	3 years
	RBT	3 years, 10 months	NA
10	BCBA	5 years, 9 months	6 months
	RBT	1 year, 1 month	NA
11	BCBA	1 year, 9 months	4 months
	RBT	1 year, 7 months	NA
12	BCBA	6 years, 9 months	2 months
	RBT	1 years, 8 months	NA
13	BCBA	5 years, 3 months	4 years
	RBT	1 year, 3 months	NA
14	BCBA	3 years, 0 months	3 years
	RBT	2 years, 1 month	NA
15	BCBA	5 years, 3 months	5 years, 3 months
	RBT	2 years	NA
16	BCBA	5 years, 3 months	5 years, 3 months
	RBT	3 years, 7 months	NA
17	BCBA	5 years, 3 months	5 years, 3 months
	RBT	4 years, 2 months	NA

All clinicians received telehealth‐specific training on how to (a) use the technology platforms for videoconferencing and client's electronic health records for data collection, (b) ensure client privacy and adhere to Health Insurance Portability and Accountability Act regulations when providing services via telehealth, (c) build rapport with families and technicians via telehealth, and (d) effectively provide coaching and feedback to the RBT working in‐person via synchronous videoconferencing. The RBTs had received prior training on how to join the videoconference for weekly clinical direction and electronic data collection when rendering in‐person direct services.

### *Participant Identification*

Archival data were obtained from a sample of 17 children and adults with ASD (ages 3 to 29 years old; average age = 11 years) who were receiving technician‐delivered in‐person services and telehealth supervision (i.e., a partial telehealth model) prior to COVID‐19 pandemic and transitioned to a full telehealth service model. Participants were included in the analysis if they transitioned from in‐person treatment delivered by an RBT with telehealth supervision to any model of full telehealth treatment (see data extraction procedures section below). Clinicians collaborated with the family and used their clinical judgement based on their knowledge of the client and prior performance on goals to select the specific telehealth service delivery model. Thus, many individuals served by the organization did not transition to a direct telehealth treatment model (i.e., they continued the pre‐COVID‐19 model, paused services) and were not included in the analysis.

All participants were receiving in‐person treatment delivered by an RBT with real‐time clinical direction via synchronous videoconferencing modalities prior to COVID‐19. Data from this type of service were needed to conduct the comparison analysis. Participants were not included in this analysis if they were new to services during COVID‐19 or immediately prior to COVID‐19 and had no in‐person comparison data available. Initially, 24 participants were identified for the archival analysis. Seven participants were excluded from the analysis because in‐person session data were not available for comparison. The in‐person data were unavailable because (a) the participant was new to treatment (n = 3), (b) the participant received new and modified goals for telehealth services (n = 1), (c) the clinician initiated a previously planned fading of treatment intensity (n = 1), (d) the participant had less than three sessions of telehealth data due to use of a combined treatment model (i.e., direct in‐person services combined with direct telehealth) (n = 1), and (e) the parent re‐initiated in‐person services (n = 1).

### *Data Extraction Procedures*

Each participant's electronic record was reviewed by the supervising BCBA. Electronic records were stored in a Health Insurance Portability and Accountability Act‐compliant, cloud‐based practice management software system (i.e., CentralReach®) that included all data, graphs, and progress notes. Records included the client's treatment history, initial assessment, treatment goals, individual targets, daily session data for skills in acquisition and behavior reduction goals, and updated progress reports. Ethnicity, diagnosis, and severity level of ASD, family household, and healthcare plan information were obtained through a review of each participant's records.

### *Child, Family, and Provider Demographics*

Participants included in the analysis were 17 clients aged 3‐17 years (35% female, 65% male). Table 2 displays participant demographics. All participants had an evaluation or confirmation of ASD diagnosis within the last 52 months (range, 9 ‐ 52 months). The primary language spoken in the home was English (n = 14), and the majority of participants were Hispanic (n = 10). Regarding group composition, 47% were from a two‐parent household, 47% from a one‐parent household, and 59% had at least one sibling. The majority of participants were receiving treatment through the state Medicaid plan (n = 15) with 71% living an average of 96 miles (range, 46 ‐ 175) from the nearest metropolitan city (n = 12). The majority of these families (75%) were required to travel across state lines to access specialty care in the nearest metropolitan city.

**Table 2:** *Participant Demographics*

Part #	Age (years)	Language	Ethnicity	Diagnosis	Gender	Household	Distance	Healthplan
1^c^	3	Spanish	Hispanic	ASD^a^	Female	2P, 1S	64	Medicaid
2	5	English	White	ASD, Level 3	Male	2P, 0S	64	Medicaid
3	5	Spanish	Hispanic	ASD, Level 3	Male	2P, 1S	100^b^	Medicaid
4	5	English	White/black	ASD, Level 1	Female	1P, 0S	0	Medicaid
5	6	English	Hispanic	ASD, Level 2	Male	1P, 1S	46^b^	Medicaid
6	7	English	Hispanic	ASD, Level 1	Male	2G, 0S	89^b^	Medicaid
7	8	English	Hispanic	ASD, Level 1	Female	1P, 2S	95^b^	Medicaid
8^c^	7	Spanish	Hispanic	ASD, Level 2	Male	2P, 1S	64	Medicaid
9	8	English	White	ASD, Level 1	Female	2P, 1S	0	Commercial
10	10	English	White	ASD, Level 2	Male	1P, 2G	175^b^	Medicaid
11	10	English	White	ASD, Level 1	Male	2P, 3S	90^b^	Medicaid
12	11	English	Hispanic	ASD, Level 3	Male	1P, 0S	0	Medicaid
13	14	English	Hispanic	ASD, Level 1	Male	2P, 1S	0	Commercial
14	19	English	Hispanic	ASD^a^	Male	1P, 4S	0	Medicaid
15	19	English	Hispanic	ASD, Level 3	Female	1P, 1S	166^b^	Medicaid
16	20	English	White	ASD, Level 2	Male	1P, 0S	108^b^	Medicaid
17	28	English	White	ASD^a^	Female	NA	89^b^	Medicaid

*Note*. Part. = Participant, ASD = Autism Spectrum Disorder. Levels refer to the levels of support per Diagnostic and Statistical Manual, fifth edition DSM‐V (Level 1 = Requiring Support; Level 2 = Requiring substantial support; Level 3 = Requiring very substantial support). P = Parent, G = Grandparent, S = Sibling

^a^ Participants were diagnosed using the DSM‐IV.

^b^ Participants must cross state lines to access specialty healthcare in the nearest metropolitan city.

^c^ Participants are siblings.

### *Participant Appropriateness Indicators*

#### *CASP‐Recommended Prerequisites*

Existing data in the client's records were reviewed for evidence of nine recommended prerequisite skills for direct telehealth treatment outlined in the CASP *Practice Parameters for Telehealth‐implementation of Applied Behavior Analysis: Continuity of Care During the COVID‐19 Pandemic* (hereafter referred to as *CASP Practice Parameters*; CASP, 2020b). The prerequisite skills included: (a) basic joint attention skills, (b) basic discrimination skills, (c) basic echoic skills, (d) basic motor imitation skill, (e) ability to follow common one‐step directions, (f) ability to participate in session with limited caregiver assistance, (g) ability to sit independently at a computer or tablet for 8‐10 min, (h) safety concerns and challenging behavior are low and/or caregivers are safely and effectively able to manage any challenging behavior, and (i) client is compliant with instructions and prompts delivered by the technician via synchronous videoconferencing and by the caregiver, if needed. Participants were scored as having evidence of the skill if they demonstrated the skill during prior clinical observations and/or via formal testing, as reflected in the archival record.

Table 3 lists the *CASP Practice Parameters* recommended prerequisite skills demonstrated by participants who transitioned to a telehealth direct service delivery model. (CASP, 2020b). Seventy six percent (n = 13) of participants demonstrated all of the recommended prerequisite skills. The majority of participants demonstrated seven of the nine prerequisite skills and indicators. Basic discrimination skills and basic motor imitation skills were evident for 100% of participants. Basic joint attention skills, basic echoic skills, ability to follow common one‐step instructions, and compliance with technician and/or caregiver prompts were each evident for 94% of participants. Additionally, 100% of participants either had low levels of challenging behavior or the caregiver was able to safely manage their child's challenging behavior.

**Table 3:** *CASP Suggested Participant Appropriateness Indicators*

Part.	Joint	Disc.	Echoic	Imit.	1‐step	Support	Attend	Prob Bx	Comp.	Total
1	✓	✓	✓	✓	✓	*–*	*–*	✓	✓	78%
2	✓	✓	✓	✓	✓	✓	✓	✓	✓	100%
3	✓	✓	✓	✓	*–*	*–*	*–*	✓	✓	67%
4	✓	✓	✓	✓	✓	✓	✓	✓	✓	100%
5	✓	✓	✓	✓	✓	*–*	*–*	✓	✓	78%
6	✓	✓	✓	✓	✓	✓	✓	✓	✓	100%
7	✓	✓	✓	✓	✓	✓	✓	✓	✓	100%
8	✓	✓	✓	✓	✓	✓	✓	✓	✓	100%
9	✓	✓	✓	✓	✓	✓	✓	✓	✓	100%
10	✓	✓	✓	✓	✓	✓	✓	✓	✓	100%
11	✓	✓	✓	✓	✓	✓	✓	✓	✓	100%
12	✓	✓	✓	✓	✓	✓	✓	✓	✓	100%
13	*–*	✓	*–*	✓	✓	*–*	*–*	✓	*–*	44%
14	✓	✓	✓	✓	✓	✓	✓	✓	✓	100%
15	✓	✓	✓	✓	✓	✓	✓	✓	✓	100%
16	✓	✓	✓	✓	✓	✓	✓	✓	✓	100%
17	✓	✓	✓	✓	✓	✓	✓	✓	✓	100%

*Not*e. Part. = participant. Joint = basic joint attention skills. Disc. = basic discrimination skills. Echoic = basic echoic skills. Imit. = basic motor imitation. 1‐step = ability to follow common 1‐step directions. Support = ability to participate with limited caregiver assistance. Attend = ability to sit independently at a computer or tablet for 8‐10 min. Prob Bx = safety concerns and challenging behavior are low and/or caregivers are safely and effectively able to manage any challenging behavior. Comp. = client is compliant with instructions and prompts delivered by the technician via synchronous videoconferencing and by the caregiver.

### *Skill Acquisition*

The clinician completed a review of medical records and treatment data pre‐ and posttelehealth direct sessions using a written task analysis with a visual job aid for extracting the data. Clinicians were instructed to enter the data into a spreadsheet using a template and a sample file for guidance.

#### *Skill Target Inclusion Criteria*

Skill acquisition data were included for a given target if there was at least one pretransition session for the target in acquisition during in‐person services and at least three sessions for telehealth direct sessions (i.e., posttransition data). We also included program data if the individual met the mastery criterion in fewer than three sessions posttransition across both in‐person and telehealth sessions (i.e., last two data points in‐person and first data point via telehealth were all scored as 100%). We analyzed data for up to five sessions pre‐and posttransition, if five sessions of data were available. We only included targets in acquisition that were measured using a percentage correct measurement system, which was 96% of skills targeted.

Information about general skill domains initially taught during direct telehealth services for each participant is provided as supplemental material on the publisher's website. The majority of programs selected for implementation were related to language skills, social skills, coping and tolerance, and adaptive living skills. The clinicians selected these programs to address the most socially significant goals and key priority areas of the clients and their families during the COVID‐19 crisis. Participants initially began with a high number of targets in generalization or maintenance phases (*M* = 55%; range, 16% ‐ 86%). This high proportion of generalization and maintenance targets was programmed specifically as an antecedent procedure to minimize challenging behavior and increase contact with reinforcement during sessions. New targets were introduced during direct telehealth services for many participants; however, these data were not included in the current analysis because in‐person sessions with those targets were not available for comparison.

#### *Service Delivery Models*

Each target taught during a telehealth session was coded to determine what type of telehealth model was used to teach the skill. All telehealth service models involved an RBT providing instruction via videoconferencing to either the child or caregiver. The main difference between models was the amount of caregiver support required. Targets were coded as (a) *technician‐delivered telehealth service model* if the client participated independently via synchronous videoconferencing without caregiver support, (b) *caregiver‐assisted telehealth model*, if the RBT delivered the treatment via videoconference and the caregiver assisted with prompting as needed, but the caregiver was not the primary intervention agent responsible for delivering treatment, and (c) a *caregiver‐implemented telehealth model* if the RBT guided the caregiver to implement all components of the intervention. Most clients received a combination of telehealth service delivery models throughout treatment. For example, some clients participated in technician‐delivered telehealth treatment for most targets but moved to a caregiver‐implemented telehealth model for adaptive living skills.

#### *Treatment Phase*

Skill acquisition targets were coded and assigned to one of three categories to gain a better understanding of the treatment phase since the expected behavior change varied based on the different types of treatment trials. Targets were coded as (a) *in acquisition* if the client had not yet demonstrated mastery of the skills as defined by their individual treatment plan (e.g., three consecutive sessions at 80% or better); (b) *generalization* if the client had met the initial mastery criteria for the target, but had not yet demonstrated the ability to perform the skill under different conditions; or (c) *maintenance* if the client had demonstrated stimulus and response generalization and the behavior was being monitored for continued demonstration of the skill.

#### *Outcomes*

The average percentage of correct independent responding for all skill acquisition targets was calculated separately for in‐person and each telehealth direct service model. We then categorized the participant's overall outcome as ‘same,’ ‘improved,’ or ‘worsened’ for each target. In order to establish an objective labeling criterion that accounts for typical performance variations across sessions, we operationally defined participant responses as *same* if their average correct independent responding during telehealth sessions was within half of one standard deviation of their in‐person sessions. Average responding that fell above this range was coded as *improved* and responding that fell below this range was coded as *worsened*. As a result, clinically nonrelevant changes in performance (e.g., 84% responding during in‐person sessions and 83% during telehealth sessions) were coded as ‘same’ since this difference was likely not outside of their expected session variation. Furthermore, the use of half of one standard deviation resulted in a highly conservative labeling criteria, which ensured that performance was not overestimated. Last, we calculated the percentage of targets that were the coded as the same or improved by dividing the number of targets labeled as same/improved outcomes by the total number of targets and multiplying by 100.

#### *Intercoder Agreement*

Intercoder agreement was completed for 33% (range, 23% ‐ 50%) of skill acquisition targets across all participants. A second independent coder was trained to review the data using the same procedures described above. In addition, the independent coder and first author reviewed data jointly and reached consensus (i.e., 100% agreement) on the data analysis during one training session. We used a point‐by‐point comparison method and scored an agreement if the session data matched exactly and a disagreement if session data did not match for each target. Next, we calculated the percentage by dividing the agreements by the agreements plus disagreements and multiplying by 100. Agreement averaged 99% (range, 96% ‐ 100%).

## Results

Table 4 provides information on the duration and dosage of services and the technology used for each participant. The average treatment duration in the partial in‐person telehealth model prior to transitioning to the direct treatment telehealth model was 20 months (range, 5 ‐ 40 months). Notably, 12 participants maintained the same or an increased dosage of ABA treatment when delivered via telehealth, with just five participants receiving a reduced treatment dosage. The dosage of in‐person treatment for participants averaged 12 hr per week (range, 0 ‐ 25), and the dosage following the transition to telehealth direct treatment averaged 11 hr per week (range, 5 ‐ 18). The transition to a telehealth service model occurred within an average of 10 days (range, 0 ‐ 55) for all participants. Families used smartphones (n = 3), tablets (n = 8), computers (n = 4), or a combination of devices (n = 2) to participate in telehealth sessions. Of those families, 18% (n = 3) were provided with technology and/or Wi‐Fi to access care.

**Table 4:** *Duration and Dosage of Services & Technology*

Part. #	Duration of Prior ABA Services (months)	Dosage/week (hours) In‐person	Dosage/week (hours) All Telehealth	Latency (days)	Technology Device
1^a^	25	15.75	5	30^b^	Smartphone, Computer
2	30	5	5	4	Tablet
3^a^	9	20	10	4	Smartphone
4	5	18	15	14^b^	Computer
5^a^	10	15	15	4	Tablet
6	14	10	10	1	Tablet
7	8	10	10	4	Smartphone
8	40	9	5	55^d^	Smartphone, Computer
9	14	0	10	10	Tablet
10	36	9	9	11	Computer
11	11	10	10	0	Tablet
12	24	9	9	16	Tablet
13^a^	12	25	15	16^b^	Tablet
14	36	8	10^c^	0	Smartphone
15	24	13	18	0	Computer
16	32	9	9	0	Tablet
17	5	18	18	2	Computer

*Not*e. Part. = Participant

^a^ Participant did not demonstrate all the CASP Suggested Participant Appropriateness Indicators.

^b^ The family or RBT had possible exposure to COVID‐19 and a14‐day quarantine was initiated.

^c^ Participant was receiving in‐person and direct telehealth services (i.e., telehealth services were implemented as part of the participant's contingency plan to promote continuity of care in the event of quarantine).

^d^ The participant was located in a community with an extreme provider shortage prior to COVID‐19. Services were only re‐initiated due to the ability to provide direct telehealth services as a local technician was still not available.

**Figure 1:**
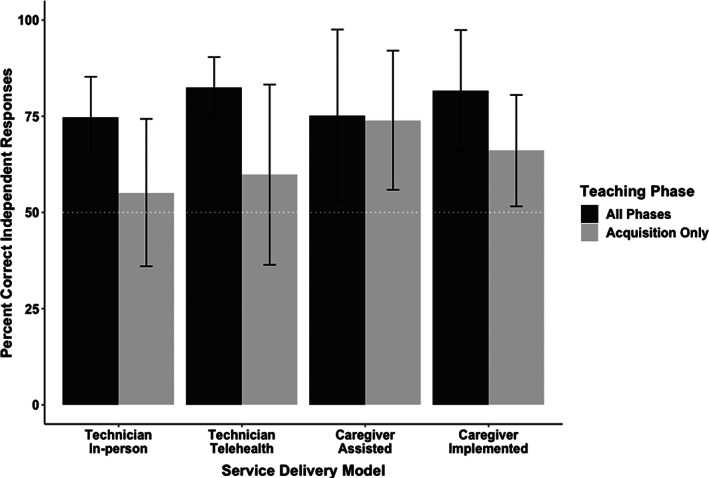
*Average Percentage of Correct Independent Responding across Participants during all Phases of Teaching and During the Acquisition Phase Only*

Figure 1 shows the average session percentage of correct independent responding for targets in all phases of teaching (i.e., targets in acquisition, generalization, and maintenance phases of teaching) and in acquisition‐only for each service delivery model. Analysis of targets in all phases of teaching indicate the average correct independent responding was 75% (SD = 11%) for in‐person direct services, 82% (SD = 8%) for telehealth direct, 75% (SD = 22%) for caregiver‐assisted telehealth, and 82% (SD = 16%) for caregiver‐implemented services. Overall average across all telehealth models was 80%. An analysis of variance (ANOVA) was used to test the null hypothesis that no difference in average session performance was observed between each service delivery model. The results of this analysis failed to reject the null hypothesis, indicating that average session percentage correct independent responding for skill acquisition targets was similar across each service delivery model (*F*(1,3) = 1.02, *MSE* = 205.7, *p* = .39).

For targets in the acquisition phase of teaching (i.e., generalization and maintenance targets were removed from the analysis), average correct independent responding was 55% (SD = 19%) for in‐person direct services, 60% (SD = 23%) for telehealth direct, 74% (SD = 18%) for caregiver‐assisted telehealth, and 66% (SD = 14%) for caregiver‐implemented services. Overall average across all telehealth models was 65%. Similar to the analysis reported above, results of an ANOVA indicated that session performance was similar across service delivery models (*F*(1,3) = 1.83, *MSE* = 735.0, *p* = .16).

**Figure 2:**
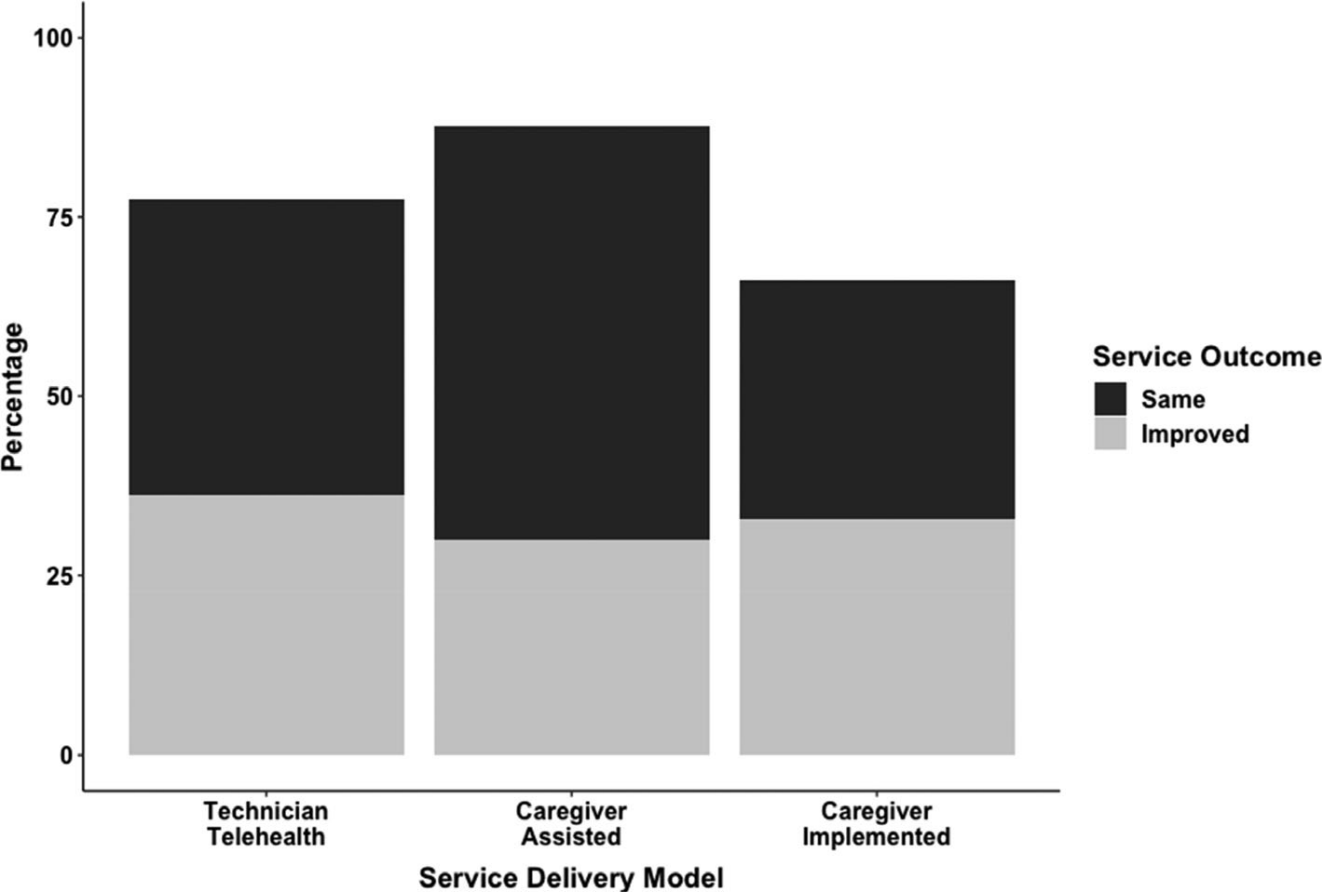
*Percentage of Targets in Acquisition with Same or Improved Performance across Participants*

Figure 2 depicts the percentage of targets in acquisition with the same or improved performance for each telehealth service delivery model when compared to in‐person direct services. Overall, 77% of acquisition targets had the same (41%) or improved (36%) performance in the direct telehealth service model, 88% of acquisition targets had the same (58%) or improved (30%) performance in the caregiver‐assisted model, and 66% of acquisition targets had the same (33%) or improved (33%) performance in the caregiver‐implemented model.

Outcome data for individual participants is available as supplementary material on the publisher's website. Eleven participants (65%) received a combination of telehealth service delivery models during the time period of this analysis (e.g., participant may have required caregiver assistance with adaptive living skills). Therefore, correct independent responding and the number of targets for each service delivery model are reported. Participants who did not demonstrate the CASP‐recommended prerequisite skills (n = 4) (as noted in the pretransition client records) primarily participated in a caregiver‐implemented service model. In comparison, participants who demonstrated all prerequisite skills immediately participated in a subset of programs via telehealth direct service delivery, with some caregiver assistance for a subset of targets. For those participants, an average of 82% of targets was delivered via direct service, 17% required caregiver assistance, and 1% were caregiver‐implemented. One participant (P14) received both in‐person and telehealth direct delivery sessions.

When compared to in‐person services, 12 participants maintained or improved performance (average increase = 10%; range, 0% ‐ 16%) across all targets in in all phases of teaching. The remaining five participants had a decrease in performance (average reduction = 7%; range, 3% ‐ 9%). Similarly, 12 participants maintained or improved performance for targets in the acquisition phase of teaching across all telehealth models (average increase = 17%; range, 0% ‐ 32%) and only five participants demonstrated a decrease in performance (average = 8%; range, 1% ‐ 16%).

## Discussion

Providers have been faced with a rapid transition from in‐person service delivery models of care to telehealth service delivery models to support families while maintaining social distancing during the COVID‐19 pandemic. To date, only two studies have been published on the direct service delivery of ABA treatment video synchronous videoconference, with participants indicating high levels of satisfaction with the treatment and positive outcomes (Ferguson et al., 2020; Pellegrino & DiGennaro‐Reed, 2020). The current findings provide further evidence of the effects of direct delivery of ABA treatment through synchronous telehealth modalities (i.e., videoconferencing) to individuals with ASD. The participants ranged in age from 3 to 28 years and the targeted skills spanned domains from social, language, and early learning repertoires to independent adaptive behavior skills.

Ongoing in‐person ABA services were successfully transitioned to telehealth services, with minimal gaps in care (*M* = 10 days; range, 0 ‐ 55) for individuals diagnosed with ASD. Notably, we were able to reestablish care for one participant (P8) who was experiencing a significant disruption in care prior to COVID‐19 due to living in a rural community with a severe provider shortage. Importantly, the results from just the initial sessions of direct telehealth treatment demonstrate maintained or improved responding for targets that were still in acquisition across all telehealth models for all participants (in‐person *M* = 55%, telehealth *M* = 65%). Moreover, participants retained a similar dosage of treatment services via telehealth (*M* = 11 hr/week) when compared to their prior in‐person service dosage (*M* = 12 hr/week).

The current investigation also provides preliminary information about the relevance of the *CASP Practice Parameters* recommended prerequisite skills and characteristics for direct treatment via telehealth (CASP, 2020b). The practice parameters had to be issued in the absence of empirical verification of the necessity of the skills; thus, this study provides an initial examination of success for individuals who demonstrated most or all of the identified skills. This archival analysis was conducted after assignment to a telehealth service delivery model. Clients included in this analysis demonstrated an average of 92% of the nine CASP‐recommended prerequisite skills and characteristics.

Those with the fewest recommended skills and characteristics (P1, P3, P5, P13) were assigned to the caregiver‐implemented model and experienced an average of 8 hr per week reduction in service delivery hours (range, 0‐11). The remaining participants averaged the same dosage of treatment posttransition (i.e., 10 hr per week). The reduced hours associated with the caregiver‐implemented model may have been due in part to these participants receiving a higher dosage of services pretransition (*M* = 19 hr/week; range, 15 ‐ 25 hr/week) compared to those participants who demonstrated all the CASP‐recommended prerequisite skills (*M* = 10 hr/week; range, 0 ‐ 18 hr/week). Caregiver responsibilities also prevented parents from providing the full dosage of treatment (e.g., employment and sibling childcare). Clinicians did not have access to the CASP‐recommended prerequisite skills during this transition and utilized their clinical judgment and family preferences to select an appropriate telehealth model. Despite the natural variability in treatment (e.g., modified treatment programs, gaps in care, dosage differences) that occurred as a result of the unplanned transition to a direct telehealth service delivery model, these results suggest that clinicians were able to provide continuity of care to families and participants who were successful in continuing to maintain and/or learn skills across all models.

While the majority of clients successfully transitioned to technician‐delivered telehealth services, the data for two clients who were not as successful were not included in the analysis. For example, one child was a new client who was about to begin treatment sessions in‐person. This client exhibited challenging behavior and the parent and technician did not have instructional control via the technician‐delivered telehealth modality. This resulted in the recommendation of a caregiver‐implemented telehealth model, which was declined due to caregiver and work responsibilities. The second participant also required a caregiver‐implemented telehealth model, but the parent was unable to provide this support given other home responsibilities; this resulted in the participant returning to in‐person services.

The heterogeneity of the group may have affected skill acquisition outcomes; however, the heterogeneity of the group can also be viewed as a strength of the findings. These preliminary results suggest that a diverse population of participants with autism with a wide range of prior experience with ABA treatment (range 5 ‐ 40 months) can benefit from some type of technician‐delivered telehealth service delivery model. Gaining an understanding of heterogeneity of treatment effect is critical for clinicians to evaluate how well a treatment can be expected to work for a subset of the population (Hagopian, 2020; Varadhan et al., 2013). Future research should continue to assess the heterogeneity of treatment effects to further our knowledge of subsets of the ASD population that may respond positively to technician‐delivered telehealth services. For example, client characteristics such as diagnostic level or the presence of certain prerequisite skills may help inform clinicians which clients will likely benefit from direct telehealth service delivery models for clients.

Several limitations should be noted. This analysis captured the available skill acquisition data for clients who transitioned from one model to the other. However, clients were not randomly assigned to this condition and there was no explicit control condition. Thus, we cannot be certain what level of outcome would have been achieved if in‐person services had continued. Due to the extraordinary circumstances brought by the COVID‐19 crisis, the analysis was restricted to outcomes achieved under the direct telehealth treatment model compared to those achieved under the prior in‐person direct model. Follow‐up data also were not collected due to time needed for long‐term evaluation of the direct telehealth service delivery model. Conclusions regarding the relevance of the recently published practice parameters (CASP, 2020b) also are difficult to draw because the analysis did not include a control group of clients who did not have the recommended skills and characteristics; it would be seemingly contraindicated to have done so. The lack of data for individuals without many of the skills means that we cannot fully evaluate whether these skills are truly prerequisites. The current data should be interpreted as providing preliminary evidence that the presence of these skills may be associated with success in the direct treatment telehealth services.

Additional research is needed to investigate the direct implementation of ABA treatment via both synchronous and asynchronous telehealth modalities, including optimal session timing, dosage, and structure. Comparison of in‐person services, partial in‐person telehealth services, and each direct telehealth service delivery is also needed in our field. Research should evaluate the optimal dosage of direct treatment and clinical oversight to guide health insurance funders on best practices and ensure optimal client outcomes (Lindgren et al., 2016; Lindgren et al., 2020; Schieltz & Wacker, 2020). Due to the fact that this was an archival analysis and data represent what occurred immediately following a public health emergency, we did not obtain social validity data from the families whose children were included in the analysis. Social validity data on family preferences and satisfaction with the different telehealth services delivery models should be gathered in future research.

Additional research also is needed to identify strategies for assessing and preteaching identified prerequisite skills before an individual completely benefits from a full telehealth service delivery model. For example, some clients at this agency initially were not considered candidates for telehealth services based on prior observations of their skills. However, direct testing of the CASP prerequisite skills revealed that those individuals demonstrated these skills, and they subsequently successfully participated in limited telehealth direct service delivery sessions. Therefore, it will be critical to formally assess a client's skills to determine baseline levels and preteach necessary prerequisite skills. As the field continues to implement treatment directly via telehealth, clinical research will be needed to further identify optimal programs and skills to teach through this modality.

Prior to the COVID‐19 pandemic, the healthcare industry was leveraging technology to address health access disparities (Rutledge et al., 2014; Spencer et al., 2020; Ward et al., 2015) and telehealth services are likely to become more widely integrated into our healthcare system (Lee et al., 2020). Telehealth affords the ability to bring specialty care knowledge to rural or medically underserved areas (Lindgren et al., 2016). Given the shortage of BCBAs and RBTs in many locations across the United States and the world, telehealth offers the ability to increase timely access to care for families who may not otherwise be able to do so. Faced with extensive travel, many families go without care or endure extensive wait times to access ABA treatment. By incorporating a partial in‐person or full telehealth service delivery model, families may have improved access to treatment (Iacono et al., 2016; Lindgren et al., 2016; Mello et al., 2016). Furthermore, without requiring the BCBA to travel for clinical oversight, the analyst may be able to reallocate their time to more clients, thus expanding provider capacity. Moreover, telehealth options may further improve provider satisfaction and reduce provider burn‐out that is common in the field ABA due to extensive travel between client homes (Gibson et al., 2009; Griffith et al., 2014; Plantiveau et al., 2018).

The COVID‐19 pandemic drastically changed the way healthcare is delivered and may have enduring impacts on healthcare service delivery. Although additional research is needed, behavior analysts leveraged technology and their knowledge of the basic principles and methodologies of ABA to continue to deliver medically necessary care to clients during this crisis. This study offers initial evidence of the promising effects of direct treatment via telehealth practice in ABA treatment.

## Supporting information


**Appendix S1:** Supporting information

## References

[jaba803-bib-0001] Akemoglu, Y. , Muharib, R. , & Meadan, H. (2020). A systematic and quality review of parent‐implemented language and communication interventions conducted via telepractice. Journal of Behavioral Education, 29, 282‐316. 10.1007/s10864-019-09356-3

[jaba803-bib-0002] Barkaia, A. , Stokes, T. F. , & Mikiashvili, T. (2017). Intercontinental telehealth coaching of therapists to improve verbalizations by children with autism. Journal of Applied Behavior Analysis, 50(3), 582–589. 10.1002/jaba.391 28436542

[jaba803-bib-0003] Barretto, A. , Wacker, D. P. , Harding, J. , Lee, J. , & Berg, W. K. (2006). Using telemedicine to conduct behavioral assessments. Journal of Applied Behavior Analysis, 39(3), 333–340. 10.1901/jaba.2006.173-04 17020213 PMC1702392

[jaba803-bib-0004] Bearss, K. , Burrell, T. L. , Challa, S. A. , Postorino, V. , Gillespie, S. E. , Crooks, C. , & Scahill, L. (2018). Feasibility of parent training via telehealth for children with autism spectrum disorder and disruptive behavior: A demonstration pilot. Journal of Autism and Developmental Disorders, 48(4), 1020–1030. 10.1007/s10803-017-3363-2 29170938

[jaba803-bib-0005] Benson, S. S. , Dimian, A. F. , Elmquist, M. , Simacek, J. , McComas, J. J. , & Symons, F. J. (2018). Coaching parents to assess and treat self‐injurious behaviour via telehealth: Self‐injurious behaviour via telehealth. Journal of Intellectual Disability Research, 62(12), 1114–1123. 10.1111/jir.12456 29205605 PMC6540986

[jaba803-bib-0006] Boisvert, M. , Lang, R. , Andrianopoulos, M. , & Boscardin, M. L. (2010). Telepractice in the assessment and treatment of individuals with autism spectrum disorders: A systematic review. Developmental Neurorehabilitation, 13(6), 423–432. 10.3109/17518423.2010.499889 20887200

[jaba803-bib-0007] Council for Autism Service Providers (2020a). *Organizational guidelines and standards: Telehealth chapter* . Retrieved from https://casproviders.org/wp-content/uploads/2020/03/CASP_ALS_Booklet_v1_1.3.20.pdf

[jaba803-bib-0008] Council of Autism Service Providers . (2020b). Practice parameters for telehealth‐implementation of applied behavior analysis: Continuity of care during COVID‐19 pandemic. Author.

[jaba803-bib-0009] Ferguson, J. , Craig, E. A. , & Dounavi, K. (2019). Telehealth as a model for providing behaviour analytic interventions to individuals with autism spectrum disorder: A systematic review. Journal of Autism and Developmental Disorders, 49(2), 582–616. 10.1007/s10803-018-3724-5 30155578 PMC6373531

[jaba803-bib-0010] Ferguson, J. L. , Majeski, M. J. , McEachin, J. , Leaf, R. , Cihon, J. H. , Leaf, J. B. (2020). Evaluating discrete trial teaching with instructive feedback delivered in a dyad arrangement via telehealth. Journal of Applied Behavior Analysis, 53(4), 1876‐1888. 10.1002/jaba.773 32914409

[jaba803-bib-0011] Fisher, W. W. , Luczynski, K. C. , Hood, S. A. , Lesser, A. D. , Machado, M. A. , & Piazza, C. C. (2014). Preliminary findings of a randomized clinical trial of a virtual training program for applied behavior analysis technicians. Research in Autism Spectrum Disorders, 8(9), 1044‐1054. 10.1016/j.rasd.2014.05.002

[jaba803-bib-0012] Gibson, J. A. , Grey, I. M. , & Hastings, R. P. (2009). Supervisor support as a predictor of burnout and therapeutic self‐efficacy in therapists working in ABA schools. Journal of Autism and Developmental Disorders, 39(7), 1024–1030. https://doir.org/10.1007/s10803-009-0709-4 19291383 10.1007/s10803-009-0709-4

[jaba803-bib-0013] Griffith, G. , Barbakou, A. , & Hastings, R. (2014). Coping as a predictor of burnout and general health in therapists working in ABA schools. European Journal of Special Needs Education, 29(4), 548–558. 10.1080/08856257.2014.952915

[jaba803-bib-0014] Hagopian, L. P. (2020). The consecutive controlled case series: Design, data‐analytics, and reporting methods supporting the study of generality. Journal of Applied Behavior Analysis, 53(2), 596‐619. 10.1002/jaba.691 32125716 PMC8805508

[jaba803-bib-0015] Hall, S. S. , Monlux, K. D. , Rodriguez, A. B. , Jo, B. , & Pollard, J. S. (2020). Telehealth‐enabled behavioral treatment for problem behaviors in boys with fragile X syndrome: a randomized controlled trial. Journal of Neurodevelopmental Disorders, 12(1), 1‐15. 10.1186/s11689-020-09331-4 33218305 PMC7679978

[jaba803-bib-0016] Health Information Technology (HIT) . (2017, September 28th). *Telemedicine and Telehealth* . https://www.healthit.gov/topic/health-it-initiatives/telemedicine-and-telehealth.

[jaba803-bib-0017] Higgins, W. J. , Luczynski, K. C. , Carroll, R. A. , Fisher, W. W. , & Mudford, O. C. (2017). Evaluation of a telehealth training package to remotely train staff to conduct a preference assessment. Journal of Applied Behavior Analysis, 50 *(* 2), 238–251. 10.1002/jaba.370 28090644

[jaba803-bib-0018] Iacono, T. , Dissanayake, C. , Trembath, D. , Hudry, K. , Erickson, S. , & Spong, J. (2016). Family and practitioner perspectives on telehealth for services to young children with autism. The Promise of New Technologies in an Age of New Health Challenges, 231, 63‐73. 10.3233/978-1-61499-712-2-63. 27782017

[jaba803-bib-0019] Ingersoll, B. , Wainer, A. L. , Berger, N. I. , Pickard, K. E. , & Bonter, N. (2016). Comparison of a self‐directed and therapist‐assisted telehealth parent‐mediated intervention for children with ASD: A pilot RCT. Journal of Autism and Developmental Disorders, 46(7), 2275–2284. 10.1007/s10803-016-2755-z 26922192

[jaba803-bib-0020] LeBlanc, L. A. , Lazo‐Pearson, J. F. , Pollard, J. S. , & Unumb, L. S. (2020). The role of compassion and ethics in decision‐making regarding access to applied behavior analysis services during the COVID‐19 crisis: A response to Cox, Plavnick & Brodhead. Behavior Analysis in Practice, 34(3), 604‐608. 10.1007/s40617-020-00446-7 PMC7296895 32837697

[jaba803-bib-0021] Lee, N. T. , Karsten, J. , & Roberts, J . (2020, May 6^th^). Removing regulatory barriers to telehealth before and after COVID‐19. Brookings Institution Retrieved May 10^th^ from: https://www.brookings.edu/research/removing-regulatory-barriers-to-telehealth-before-and-after-covid-19/

[jaba803-bib-0022] Lerman, D. C. , O'Brien, M. J. , Neely, L. , Call, N. A. , Tsami, L. , Schieltz, K. M. , Berg, W. K. , Graber, J. , Huang, P. , Kopelman, T. & Cooper‐Brown, L. J. (2020). Remote coaching of caregivers via telehealth: Challenges and potential solutions. Journal of Behavioral Education, 29(2), 195‐221. 10.1007/s10864-020-09378-2 PMC9455948 36093285

[jaba803-bib-0023] Lindgren, S. , Wacker, D. , Schieltz, K. , Suess, A. , Pelzel, K. , Kopelman, T. , Lee, J. , Romani, P. , & O'Brien, M. (2020). A randomized controlled trial of functional communication training conducted via telehealth for young children with autism spectrum disorder. Journal of Autism and Developmental Disorders. Advance online publication. 10.1007/s10803-020-04451-1 PMC7572463 32300910

[jaba803-bib-0024] Lindgren, S. , Wacker, D. , Suess, A. , Schieltz, K. , Pelzel, K. , Kopelman, T. , Lee, J. , Romani, P. , & Waldron, D. (2016). Telehealth and autism: Treating challenging behavior at lower cost. Pediatrics, 137(Supplement 2), S167–S175. 10.1542/peds.2015-2851O 26908472 PMC4727312

[jaba803-bib-0025] McLay, L. , Sutherland, D. , Machalicek, W. , & Sigafoos, J. (2020). Systematic review of telehealth interventions for the treatment of sleep problems in children and adolescents. Journal of Behavioral Education, 29, 222‐245. 10.1007/s10864-020-09364-8

[jaba803-bib-0026] Megan, K . (2020, April 1^st^). During COVID‐19, disabled adults lack services and support. The Connecticut News Project. Retrieved May10th from: https://ctmirror.org/2020/04/01/during-covid-19-disabled-adults-lack-services-and-supports/.

[jaba803-bib-0027] Mello, M. P. , Goldman, S. E. , Urbano, R. C. , & Hodapp, R. M. (2016). Services for children with autism spectrum disorder: Comparing rural and non‐rural communities. Education and Training in Autism and Developmental Disabilities, 51(4), 355‐365. 10.2307/26173863.

[jaba803-bib-0028] Monlux, K. D. , Pollard, J. S. , Bujanda Rodriguez, A. Y. , & Hall, S. S. (2019). Telehealth delivery of function‐based behavioral treatment for problem behaviors exhibited by boys with Fragile X Syndrome. Journal of Autism and Developmental Disorders, 49(6), 2461–2475. 10.1007/s10803-019-03963-9 30937736

[jaba803-bib-0029] Myers, K. , Nelson, E. L. , Rabinowitz, T. , Hilty, D. , Baker, D. , Barnwell, S. S. , Boyce, G. , Bufka, L. F. , Cain, S. , & Chui, L. (2017). American telemedicine association practice guidelines for telemental health with children and adolescents. Telemedicine and E‐Health, 23(10), 779–804. 10.1089/tmj.2017.0177 28930496

[jaba803-bib-0030] Pellegrino, A. , & DiGennaro Reed, F. (2020). Using telehealth to teach valued skills to adults with intellectual and developmental disabilities. Journal of Applied Behavior Analysis, 53(3), 1276‐1289. 10.1002/jaba.743 32542669

[jaba803-bib-0032] Plantiveau, C. , Dounavi, K. , & Virués‐Ortega, J. (2018). High levels of burnout among early‐career board‐certified behavior analysts with low collegial support in the work environment. European Journal of Behavior Analysis, 19(2), 195‐207. 10.1080/15021149.2018.1438339

[jaba803-bib-0033] Pollard, J. S. , Karimi, K. A. , & Ficcaglia, M. B. (2017). Ethical considerations in the design and implementation of a telehealth service delivery model. Behavior Analysis: Research and Practice, 17(4), 298–311. 10.1037/bar0000053

[jaba803-bib-0034] Rispoli, M. , & Machalicek, W. (2020). Advances in telehealth and behavioral assessment and intervention in education: Introduction to the special issue. Journal of Behavioral Education, 29, 189‐194. 10.1007/s10864-020-09383-5

[jaba803-bib-0036] Rutledge, C. M. , Haney, T. , Bordelon, M. , Renaud, M. , & Fowler, C. (2014). Telehealth: Preparing advanced practice nurses to address healthcare needs in rural and underserved populations. International Journal of Nursing Education Scholarship, 11(1), 1‐9. 10.1515/ijnes-2013-0061 24423469

[jaba803-bib-0037] Schieltz, K. M. , & Wacker, D. P. (2020). Functional assessment and function‐based treatment delivered via telehealth: A brief summary. Journal of Applied Behavior Analysis, 53(3), 1242‐1258. 10.1002/jaba.742 32643811 PMC7361834

[jaba803-bib-0038] Spencer, T. , Noyes, E., & Biederman, J. (2020). Telemedicine in the management of ADHD: Literature review of telemedicine in ADHD. Journal of Attention Disorders, 24(1), 3‐9. 10.1177/1087054719859081 31257978

[jaba803-bib-0039] Suess, A N. , Romani, P. W. , Wacker, D. P. , Dyson, S. M. , Kuhle, J. L. , Lee, J. F. , Lindgren, S. D. , Kopelman, T. G. , Pelzel, K. E. , & Waldron, D. B. (2014). Evaluating the treatment fidelity of parents who conduct in‐home functional communication training with coaching via telehealth. Journal of Behavioral Education, 23(1), 34–59. 10.1007/s10864-013-9183-3

[jaba803-bib-0040] Suess, A. N. , Schieltz, K. M. , Wacker, D. P. , Detrick, J. , & Podlesnik, C. A. (2020). An evaluation of resurgence following functional communication training conducted in alternative antecedent contexts via telehealth. Journal of the Experimental Analysis of Behavior, 113(1), 278–301. 10.1002/jeab.551 31617951

[jaba803-bib-0041] Suess, A. N. , Wacker, D. P. , Schwartz, J. E. , Lustig, N. , & Detrick, J. (2016). Preliminary evidence on the use of telehealth in an outpatient behavior clinic. Journal of Applied Behavior Analysis, 49(3), 686–692. 10.1002/jaba.305 27001117

[jaba803-bib-0042] Tomlinson, S. R. L. , Gore, N. , & McGill, P. (2018). Training individuals to implement applied behavior analytic procedures via telehealth: A systematic review of the literature. Journal of Behavioral Education, 27(2), 172–222. 10.1007/s10864-018-9292-0

[jaba803-bib-0043] Unholz‐Bowden, E. , McComas, J. J. , McMaster, K. L. , Girtler, S. N. , Kolb, R. L. , & Shipchandler, A. (2020). Caregiver training via telehealth on behavioral procedures: A systematic review. Journal of Behavioral Education, 29(2), 246‐281. 10.1007/s10864-020-09381-7 PMC10479951 37670908

[jaba803-bib-0044] Varadhan, R. , Segal, J. B. , Boyd, C. M. , Wu, A. W. , & Weiss, C. O. (2013). A framework for the analysis of heterogeneity of treatment effect in patient‐centered outcomes research. Journal of Clinical Epidemiology, 66(8), 818–825. 10.1016/j.jclinepi.2013.02.009 23651763 PMC4450361

[jaba803-bib-0045] Vismara, L. A. , Young, G. S. , Stahmer, A. C. , Griffith, E. M. , & Rogers, S. J. (2009). Dissemination of evidence‐based practice: Can we train therapists from a distance? Journal of Autism and Developmental Disorders, 39(12), 1636–1651. 10.1007/s10803-009-0796-2 19582564 PMC2777219

[jaba803-bib-0046] Wacker, D. P. , Lee, J. F. , Dalmau, Y. C. P. , Kopelman, T. G. , Lindgren, S. D. , Kuhle, J. , Pelzel, K. E. , & Waldron, D. B. (2013a). Conducting functional analyses of problem behavior via telehealth. Journal of Applied Behavior Analysis, 46(1), 31–46. 10.1002/jaba.29 24114083 PMC5361405

[jaba803-bib-0047] Wacker, D. P. , Lee, J. F. , Padilla Dalmau, Y. C. , Kopelman, T. G. , Lindgren, S. D. , Kuhle, J. , Pelzel, K. E. , Dyson, S. , Schieltz, K. M. , & Waldron, D. B. (2013b). Conducting functional communication training via telehealth to reduce the problem behavior of young children with autism. Journal of Developmental and Physical Disabilities, 25(1), 35–48. 10.1007/s10882-012-9314-0 23543855 PMC3608527

[jaba803-bib-0048] Wainer, A. L. , & Ingersoll, B. R. (2015). Increasing access to an ASD imitation intervention via a telehealth parent training program. Journal of Autism and Developmental Disorders, 45(12), 3877–3890. 10.1007/s10803-014-2186-7 25035089

[jaba803-bib-0049] Ward, M. M. , Jaana, M. , & Natafgi, N. (2015). Systematic review of telemedicine applications in emergency rooms. International Journal of Medical Informatics, 84(9), 601‐616. 10.1016/j.ijmedinf.2015.05.009 26072326

[jaba803-bib-0050] Wolfram, J . (2020, March 30^th^). Parents worry: Will upended routine mean children with autism lose ground? Modern Kids: Coronavirus Pandemic. Retrieved May10th, 2020 from https://whyy.org/articles/parents‐worry‐will‐upended‐routines‐mean‐children‐with‐autism‐lose‐ground/

