# Supplementary material for: The effects of transition to technician‐delivered telehealth ABA treatment during the COVID‐19 crisis: A preliminary analysis
Source: J Appl Behav Anal. 2020 Dec 28;54(1):87–102. doi: 10.1002/jaba.803 (PMC7898711; doi:10.1002/jaba.803)
Supplement: Supplementary file 1 — Appendix S1: Supporting information [file JABA-54-87-s001.docx]

**Supplementary Table 1**

*Domains Targeted in Telehealth ABA Sessions for each Participant*

| Participant | Domain (# targets) | Percentage of targets in  Generalization or Maintenance Phase of Teaching | |
| --- | --- | --- | --- |
| 1 | Manding (6) | 53% | |
|  | Echoics (3) |  | |
|  | Tacting (5) |  | |
|  | Social Skills (1) |  | |
|  | Listener Skills (2)  Adaptive Living (1) |  | |
| 2 | Social Play Skills(3) | 55% | |
|  | Listener Skills (6) |  | |
|  | Coping & Tolerance (2) |  | |
| 3 | Echoic (6)  Tacting (6)  Motor Imitation (3)  Adaptive Living (2)  Coping & Tolerance (1) | 76% | |
| 4 | Manding (4)  Intraverbal skills (3) | 47% | |
|  | Listener skills (2) |  | |
|  | Adaptive Living (3) |  | |
|  | Coping & Tolerance (1)  Social Skills (8) |  | |
| 5 | Manding (8)  Listener Skills (12)  Echoic (7)  Imitation (10)  Adaptive Living (2) | 62% | |
| 6 | Manding (5)  Intraverbal skills (1)  Emotional Development (18)  Adaptive Living (1)  Coping & Tolerance (5)  Social Skills (18) | 71% | |
| 7 | Manding (1)  Coping & Tolerance (6)  Social Skills (5) | 33% | |
| 8 | Manding (1)  Tacting (4)  Visual Discrimination (1)  Coping & Tolerance (1)  Play/Social Skills (2) | 67% | |
| 9 | Manding (1)  Tacting (3)  Adaptive Living (1)  Coping & Tolerance (5)  Social Skills (7) | 62% | |
| 10 | Manding (1)  Listener skills (1)  Coping & Tolerance (5)  Social Skills (1) | 37% | |
| 11 | Social Skills (2)  Adaptive Living (3)  Coping & Tolerance (4) | 62% | |
| 12 | Manding (2)  Tacting (12)  Intraverbal (1)  Listener Skills (2)  Social Skills (5) | 68% | |
| 13 | Manding (5)  Adaptive Living (12)  Listener Skills (2)  Social Skills (1) | | 16% |
| 14 | Social Skills (4)  Adaptive Living (2)  Coping & Tolerance (1) | | 86% |
| 15 | Manding (1)  Adaptive Living (18)  Coping & Tolerance (1) | | 65% |
| 16 | Adaptive Living (8)  Coping & Tolerance (2)  Self-management (7) | | 19% |
| 17 | Social Skills (8)  Adaptive Living (1)  Coping & Tolerance (3) | | 58% |

*Note.* The percentage of total targets that were in generalization or maintenance phase is equal to 100% minus the percentage of targets in acquisition.

**Supplementary Table 2**

*Individual Participant Outcome Data*

|  | | |  | | Average % Correct Across Service Delivery Models (Targets) | | | | | | |
| --- | --- | --- | --- | --- | --- | --- | --- | --- | --- | --- | --- |
| Part # Phase | |  | | In-person  Direct | |  | Telehealth  Direct | Caregiver- Assisted | Caregiver-Implemented |  | All Telehealth Models |
| 1^a^ | All phases |  | | 81% (17) | |  | 89% (7) | 78% (2) | 68% (8) |  | 78% (17) |
|  | Acquisition |  | | 72% (8) | |  | 59% (1) | 78% (2) | 49% (5) |  | 62% (8) |
|  | Same |  | | -- | |  | 0% (0) | 50% (1) | 40% (2) |  | 38% (3) |
|  | Improved |  | | -- | |  | 0% (0) | 0% (0) | 0% (0) |  | 0% (0) |
|  |  |  | |  | |  |  |  |  |  |  |
| 2 | All phases |  | | 92% (11) | |  | 91% (6) | 84% (5) | -- |  | 88% (11) |
|  | Acquisition |  | | 85% (5) | |  | -- | 84% (5) | -- |  | 84% (5) |
|  | Same |  | | -- | |  | -- | 80% (4) | -- |  | 80% (4) |
|  | Improved |  | | -- | |  | -- | 0% (0) | -- |  | 0% (0) |
|  |  |  | |  | |  |  |  |  |  |  |
| 3^a^ | All phases |  | | 87% (18) | |  | -- | -- | 94% (18) |  | 94% (18) |
|  | Acquisition |  | | 58% (5) | |  | -- | -- | 80% (5) |  | 80% (5) |
|  | Same |  | | -- | |  | -- | -- | 40% (2) |  | 40% |
|  | Improved |  | | -- | |  | -- | -- | 40% (2) |  | 40% |
|  |  |  | |  | |  |  |  |  |  |  |
| 4 | All phases |  | | 70% (21) | |  | 83% (17) | 89% (2) | 98% (2) |  | 85% (21) |
|  | Acquisition |  | | 58% (4) | |  | 69% (4) | -- | -- |  | 69% (4) |
|  | Same |  | | -- | |  | 50% (2) | -- | -- |  | 50% (2) |
|  | Improved |  | | -- | |  | 25% (1) | -- | -- |  | 25% (1) |
|  |  |  | |  | |  |  |  |  |  |  |
| 5 ^a^ | All phases |  | | 77% (39) | |  | -- | 91% (16) | 85% (23) |  | 87% (39) |
|  | Acquisition |  | | 55% (17) | |  | -- | 81% (4) | 76% (13) |  | 78% (17) |
|  | Same |  | | -- | |  | -- | 25% (1) | 23% (3) |  | 24% (4) |
|  | Improved |  | | -- | |  | -- | 75% (3) | 62% (8) |  | 65% (11) |
|  |  |  | |  | |  |  |  |  |  |  |
| 6 | All phases |  | | 81% (49) | |  | 75% (49) | -- | -- |  | 75% (49) |
|  | Acquisition |  | | 44% (15) | |  | 28% (15) | -- | -- |  | 28% (15) |
|  | % Same |  | | -- | |  | 47% (7) | -- | -- |  | 47% (7) |
|  | Improved |  | | -- | |  | 7% (1) | -- | -- |  | 7% (1) |
|  |  |  | |  | |  |  |  |  |  |  |
| 7 | All phases |  | | 61% (12) | |  | 65% (6) | 40% (6) | -- |  | 52% (12) |
|  | Acquisition |  | | 54% (8) | |  | 60% (3) | 39% (5) | -- |  | 49% (8) |
|  | Same |  | | -- | |  | 67% (2) | 40% (2) | -- |  | 50% (4) |
|  | Improved |  | | -- | |  | 0% (0) | 20% (1) | -- |  | 13% (1) |
|  |  |  | |  | |  |  |  |  |  |  |
| 8 | All phases |  | | 72% (9) | |  | 79% (7) | 95% (2) | -- |  | 82% (9) |
|  | Acquisition |  | | 42% (3) | |  | 53% (1) | 95% (2) | -- |  | 74% (3) |
|  | Same |  | | -- | |  | 0% (0) | 50% (1) | -- |  | 33% (1) |
|  | Improved |  | | -- | |  | 100% (1) | 50% (1) | -- |  | 67% (2) |
|  |  |  | |  | |  |  |  |  |  |  |
| 9 | All phases |  | | 64% (17) | |  | 77% (13) | 76% (4) | -- |  | 77% (17) |
|  | Acquisition |  | | 41% (7) | |  | 62% (6) | 50% (1) | -- |  | 56% (7) |
|  | Same |  | | -- | |  | 50% (3) | 100% (1) | -- |  | 57% (4) |
|  | Improved |  | | -- | |  | 50% (3) | 0% (0) | -- |  | 43% (3) |
|  |  |  | |  | |  |  |  |  |  |  |
| 10 | All phases |  | | 89% (8) | |  | 84% (8) | -- | -- |  | 84% (8) |
|  | Acquisition |  | | 82% (5) | |  | 78% (5) | -- | -- |  | 78% (5) |
|  | Same |  | | -- | |  | 40% (2) | -- | -- |  | 40% (2) |
|  | Improved |  | | -- | |  | 20% (1) | -- | -- |  | 20% (1) |
|  |  |  | |  | |  |  |  |  |  |  |
| 11 | All phases |  | | 72% (9) | |  | 83% (8) | 79% (1) | -- |  | 83% (9) |
|  | Acquisition |  | | 67% (3) | |  | 62% (2) | 79% (1) | -- |  | 71% (3) |
|  | Same |  | | -- | |  | 100% (2) | 100% (1) | -- |  | 100% (3) |
|  | Improved |  | | -- | |  | 0% (0) | 0% (0) | -- |  | 0% (0) |
|  |  |  | |  | |  |  |  |  |  |  |
| 12 | All phases |  | | 79% (24) | |  | 87% (22) | 87% (2) | -- |  | 87% (24) |
|  | Acquisition |  | | 49% (7) | |  | 61% (5) | 87% (2) | -- |  | 74% (7) |
|  | Same |  | | -- | |  | 0% (0) | 0% (0) | -- |  | 0% (0) |
|  | Improved |  | | -- | |  | 60% (3) | 100% (2) | -- |  | 71% (5) |
|  |  |  | |  | |  |  |  |  |  |  |
| 13^a^ | All phases |  | | 54% (20) | |  | -- | -- | 63% (20) |  | 63% (20) |
|  | Acquisition |  | | 51% (10) | |  | -- | -- | 60% (10) |  | 60% (10) |
|  | Same |  | | -- | |  | -- | -- | 30% (3) |  | 30% (3) |
|  | Improved |  | | -- | |  | -- | -- | 30% (3) |  | 30% (3) |
|  |  |  | |  | |  |  |  |  |  |  |
| 14 | All phases |  | | 74% (7) | |  | 82% (6) | 24% (1) | -- |  | 74% (7) |
|  | Acquisition |  | | 0% (1) | |  | 0 (1) | -- | -- |  | 0% (1) |
|  | Same |  | | -- | |  | 100% (1) | -- | -- |  | 100% (0) |
|  | Improved |  | | -- | |  | 0% | -- | -- |  | 0% (0) |
|  |  |  | |  | |  |  |  |  |  |  |
| 15 | All phases |  | | 81% (20) | |  | 89% (7) | 85% (13) | -- |  | 86% (20) |
|  | Acquisition |  | | 67% (7) | |  | 85% (3) | 74% (4) | -- |  | 80% (7) |
|  | Same |  | | -- | |  | 0% (0) | 75% (3) | -- |  | 43% (3) |
|  | Improved |  | | -- | |  | 100% (3) | 25% (1) | -- |  | 57% (4) |
|  |  |  | |  | |  |  |  |  |  |  |
| 16 | All phases |  | | 59% (17) | |  | 75% (17) | -- | -- |  | 75% (17) |
|  | Acquisition |  | | 53% (14) | |  | 72% (14) | -- | -- |  | 72% (14) |
|  | Same |  | | -- | |  | 43% (6) | -- | -- |  | 43% (6) |
|  | Improved |  | | -- | |  | 50% (7) | -- | -- |  | 50% (7) |
|  |  |  | |  | |  |  |  |  |  |  |
| 17 | All phases |  | | 78% (12) | |  | 94% (12) | -- | -- |  | 94% (12) |
|  | Acquisition |  | | 59% (5) | |  | 87% (5) | -- | -- |  | 87% (5) |
|  | Same |  | | -- | |  | 40% (2) | -- | -- |  | 40% (2) |
|  | Improved |  | | -- | |  | 60% (3) | -- | -- |  | 60% (3) |
|  |  |  | |  | |  |  |  |  |  |  |

*Not*e. Part. = Participant

^a^Participant did not demonstrate all the CASP Suggested Participant Appropriateness Indicators
